# Irisin promotes the proliferation and tenogenic differentiation of rat tendon-derived stem/progenitor cells via activating YAP/TAZ

**DOI:** 10.1007/s11626-022-00699-2

**Published:** 2022-09-20

**Authors:** Langhai Xu, Zhonggai Chen, Tingting Geng, Bin Ru, Quan Wan, Jianbin Zhang, Shun Li, Wenjun Cai

**Affiliations:** 1 Department of Pain, Zhejiang Provincial People’s Hospital, People’s Hospital of Hangzhou Medical College, Hangzhou, China; 2 Department of Orthopaedics, The Second Affiliated Hospital and Yuying Children’s Hospital of Wenzhou Medical University, Wenzhou, China; 3 Shulan (Hangzhou) Hospital Affiliated to Zhejiang Shuren University Shulan International Medical College, Hangzhou, China; 4 Department of Oncology, Clinical Research Institute, Zhejiang Provincial People’s Hospital, People’s Hospital of Hangzhou Medical College, Hangzhou, China

**Keywords:** Cell differentiation, Irisin, Stem cells, Tissue engineering, YAP/TAZ

## Abstract

Tendinopathy is a common tendon disorder characterized by pain, swelling, and dysfunction. Current evidence has demonstrated that the depletion of stem cell pool and non-tenogenic differentiation of tendon-derived stem/progenitor cells (TSPCs) might account for the pathogenesis of tendinopathy. FNDC5/Irisin, as a novel exercise-induced myokine, is proved to be involved in the exercise-induced protective effects on musculoskeletal disorders. However, whether irisin can affect TSPCs fate is still unknown. To ascertain the roles of irisin on the proliferation and tenogenic differentiation of TSPCs, rat TSPCs were isolated and incubated with irisin. Cell viability, phenotypic changes, and related signaling pathways were evaluated by CCK-8 assay, colony formation assay, real-time PCR, Western blot, immunofluorescence, and proteasome activity assay. We found that irisin treatment increased the proliferative and colony-forming abilities, and promoted the tenogenic differentiation of TSPCs by upregulating the expression of YAP/TAZ. In conclusion, our work showed for the first time that irisin promotes the proliferation and tenogenic differentiation of rat TSPCs in vitro by activating YAP/TAZ, and the process was associated with a ubiquitin-proteasome proteolytic pathway. In conclusion, irisin and agents targeting YAP/TAZ may be promising therapeutic options for tendinopathy.

**Supplementary Information:**

The online version contains supplementary material available at 10.1007/s11626-022-00699-2.

## Introduction

Tendons are tough, high-tensile-strength bands of the collagenous musculoskeletal tissues that connect muscle to bone to allow for body movement. Tendinopathy is a clinical condition characterized by pain, swelling, and dysfunction, mainly arising from overuse. Among all low limb tendinopathies, Achilles tendinopathy is the most prevalent in the general population and has a prevalence of 6.2–9.5% and 11.83% in the athletic and non-athletic population respectively (Murphy *et al.* 2018), causing a severe economic and social burden. Current tendinopathy management tends to support exercise-based therapy as the most evidence-based intervention (Malliaras 2017; Rio *et al.* 2017; Cardoso *et al.* 2019). The adjuncts, such as electrophysical agents, nonsteroidal anti-inflammatory drugs, corticosteroid injections, and surgery, may be required as adjuvant therapies. However, the healing process of injured tendon is usually slow and fails to regain the complete function of the normal tendon with these treatments (Gaspar *et al.* 2015; Liu *et al.* 2017).

Tendon-resident stem cells, termed tendon-derived stem/progenitor cells (TSPCs), were firstly identified in human and mouse tendons by Bi et al. in 2007 (Bi *et al.* 2007). At present, TSPCs have also been isolated and cultured from various species, such as rabbit, rat, and equine (Rui *et al.* 2010; Yin *et al.* 2010; Zhang and Wang 2010; Durgam *et al.* 2016). Similar to other classical mesenchymal stem cells (MSCs), TSPCs share some common characteristics, including surface markers, self-colony ability, and multi-lineage differentiation potential (Zhang *et al.* 2016). Besides, TSPCs also express tendon-related markers, such as scleraxis and tendomodulin, and can differentiate into tendon-like tissues. Therefore, the viability and tenogenic differentiation of TSPCs may play a critical role in tendon maintenance and regeneration.

Irisin is a well-defined myokine derived from proteolytic cleavage of the fibronectin type III domain-containing 5 (FNDC5) gene and is secreted mainly by skeletal muscle (Boström *et al.* 2012). During exercise, peroxisome proliferator-activated receptor gamma coactivator 1α (PGC-1α) signaling transduction activates FNDC5 expression to increase the secretion of irisin (Boström *et al.* 2012). Initial studies have shown that irisin can promote muscle development and browning of white adipose tissue (Boström *et al.* 2012). Further studies have shown that irisin plays a vital role in a variety of physiological and pathological processes such as inflammation, proliferation, aging, metastasis, and neurogenesis (Panati *et al.* 2016; Rabiee *et al.* 2020). It has been mentioned above that exercise has been regarded as the most evidence-based intervention for tendinopathy (Malliaras 2017; Rio *et al.* 2017; Cardoso *et al.* 2019). Interestingly, recent study performed by Zhang et al. showed that exercise exerted its anabolic effects on tendons by increasing TSPC proliferation and collagen synthesis in mouse (Zhang *et al.* 2010). This study further confirmed the beneficial effects of exercise on TSPCs in injured tendons. However, irisin as the secretory product of skeletal muscle during exercise, whether irisin has a beneficial effect on tendon repair is still unknown.

In the present study, we investigated the relationship between irisin and the proliferation and tenogenic differentiation of TSPCs. We concentrated on the phenotypic changes of TSPCs and the underlying mechanism. We hope this work will provide a novel strategy for tendon repair.

## Materials and methods

### Reagents

Recombinant rat irisin was purchased from R&D Systems, Abingdon, UK. Verteporfin was purchased from Selleck, Shanghai, China. Dulbecco’s modified Eagle’s medium (DMEM), penicillin/ streptomycin, fetal bovine serum (FBS), and 0.25% trypsin were obtained from Gibco BRL, Grand Island, NY. Collagenase I and ascorbic acid were purchased from Sigma-Aldrich, St, Louis, MO.

### Cell culture

Achilles tendons were obtained from 3-wk-old Sprague-Dawley (SD) rats. Under sterile conditions, the tendons were cut into 1-mm^3^ particles, and the tissues were incubated with 0.1% type I collagenase at 37°C for 2h to isolate tendon cells. Single-cell tendon-derived cells were cultured in 48-well plates for 7 d, and colonies were collected as passage 0 (P0). The cells were passaged at a ratio of 1:3, and P3 cells were used. DMEM supplemented with 10% FBS and 100 units/ml penicillin and 100 μg/ml streptomycin was used to expand single-cell colonies. Cells were cultured at 37°C with 5% CO_2_. All experiments were performed with mycoplasma-free cells.

### Identification of surface markers

Cells were stained with fluorescent primary antibody on ice for 40 min, washed 3 times, and detected by flow cytometry. The negative control had no fluorescent antibody. Fluorescent primary antibody: FITC anti-rat CD29, FITC anti-rat CD44, PE anti-rat CD45, PE anti-rat CD90 (Bioleague, Poggensee, Germany).

### Cell viability assay

To analyze the toxicity of irisin on TSPCs, CCK-8 assay (Dojindo Molecular, Rockville, MD) was used in accordance with the manufacturer’s instruction. The cells were seeded into 96-well plates (5 × 10^3^/well), then treated with various concentrations of irisin for 3 d. The cells were then incubated with fresh media (containing 10% CCK-8 solution) for 2h at 37°C and read at a wavelength of 450nm with a microplate spectrophotometer.

### Colony formation assay

To analyze the effect of irisin on TSPC proliferation, the cells were seeded into 96-well plates (5 × 10^3^/well), then treated with various concentrations of irisin for 3 d. The cell colony number and size were counted and calculated under a Leica optical microscope.

### Western blot

After treatment, total cellular proteins were extracted with RIPA according to the manufacturer’s protocol. Nuclear Extraction Reagents (Bosterbio, Wu Han, China) were used to prepare nuclear extracts. Equal amounts of extracted proteins were separated via 10% sodium dodecyl sulfate (SDS)–polyacrylamide gels and transferred into nitrocellulose membranes. After being blocked with 5% BSA for 1h, the membranes were incubated with primary antibodies overnight at 4°C. Afterwards, the membranes were incubated with secondary antibodies at room temperature for 1 h and then detected with the Bio-Rad ChemiDoc System. All assays were performed in triplicate. The relative amount of proteins was analyzed with Quantity One software (Bio-Rad, Hercules, CA). GAPDH and TBP worked as endogenous control for total cellular and nuclear protein analysis respectively.

### Real-time PCR

After treatment, total RNA was extracted with TRIzol reagent (Invitrogen, Carlsbad, CA) according to the manufacturer’s instructions. Total RNA was used to synthesize cDNA by reverse transcription (cDNA synthesis kit, Takara, Kusatsu, Japan). Then, the cDNA samples were replicated using SYBR Premix Ex Taq II (Takara) by ABI StepOnePlus System. Each 10μl sample contained 5μl of SYBR Premix Ex Taq II, 0.4μl of each forward and reverse primer, 1μl of cDNA (10ng), and 3.2μl of ddH2O. The reaction conditions were as follows: denaturation, 95°C×30 s, followed by 40 cycles of 95°C×15 s→60°C×32 s→72°C×1 min→72°C×5 min. GAPDH was used as the endogenous control. Data were analyzed for fold difference Using the 2^−ΔΔCT^ method. All assays were performed in triplicate. The primer pairs shown in Table 1 were used for the real-time PCR (RT-PCR) amplification.

**Table 1:** Primer sequences used in this study

Gene	Forward	Reverse
bFGF	GGACGGCTGCTGGCTTCTAA	CCAGTTCGTTTCAGTGCCACATAC
C-MYC	ATCACAGCCCTCACTCAC	ACAGATTCCACAAGGTGC
CTGF	CCGCCAACCGCAAGATT	CACGGACCCACCGAAGAC
COL1	GAGAGCATGACCGATGGATT	CCTTCTTGAGGTTGCCACTC
SCX	AACACGGCCTTCACTGCGCTG	CAGTAGCACGTTGCCCAGGTG
TNMD	TGGGGGAGCAAACACTTCTG	TCTTCTTCTCGCCATTGCTGT
YAP	CGTGCCCATGAGGCTTCGCA	TCGGTACTGGCCTGTCGCGA
18S	CCTGAGAAACGGCTACCACA	ACCAGACTTGCCCTCCAATG

### Immunofluorescence

TSPCs cultured on 24-well plates were incubated with irisin for various times (0, 1d, 3d, 7d). After fixation with methanol, cells were permeabilized by PBS containing 0.5% v/v Triton X-100 for 20min and blocked with 5% BSA at room temperature for 2h. Cells were incubated with primary antibody against YAP at 4°C overnight, followed by being incubated with fluorescein isothiocyanate-conjugated secondary antibodies for 1h. Cell nucleuses were stained with DAPI for 5min. Then, cells were analyzed with a Leica fluorescence microscope.

### Enzyme-linked immunosorbent assay (ELISA)

The endogenous irisin secretion in cells was quantified in culture media by a commercial ELISA kit (Phoenix Pharmaceuticals, Inc.; EK-067-29) according to the manufacturer. The TSPCs were cultured in the tenogenic differentiation induction medium comprising high-glucose DMEM, 10% FBS, 37.5 μg/ml ascorbic acid, and 1% penicillin G for 7 d. The medium was changed every day. The irisin levels were detected by collecting the cell culture supernates from each sample at day 1, 3, and 7. Then, absorbance from each sample was measured at the wavelength of 450nm using a spectrophotometer, and the concentrations of FNDC5/Irisin were determined by comparing the optical density (OD) values of the tested samples to that of the standard curve. All experiments were performed three times.

### Proteasome activity assay

A Proteasome-GloTM Chymotrypsin-like Cell-Based Assays kit (Promega, Madison, WI) was used according to the manufacturer’s instructions. Briefly, TSPCs were seeded at 10,000 cells per well with 100μl in 96-well plates. After irisin treatment, an equal volume of luminogenic substrate specified for chymotrypsin-like protease activity was added to samples. After shaking for 2min and incubation at room temperature for 10min, luminescence was detected by a luminometer (BioTek Instruments, Winooski, VT). To confirm assay specificity, the same number of samples was pretreated for 1h with 10μM epoxomicin, a proteasome inhibitor. For each sample, proteasome activity was normalized with epoxomicin-pretreated luminescence as the background signal.

### Statistical analysis

All data are presented as means ± SDs. One-way ANOVA with a subsequent post hoc Tukey’s test was used for multiple comparisons. *P*<0.05 is considered statistically significant.

## Results

### Effects of irisin on the proliferative and colony-forming abilities of rat TSPCs

**Figure 1.**
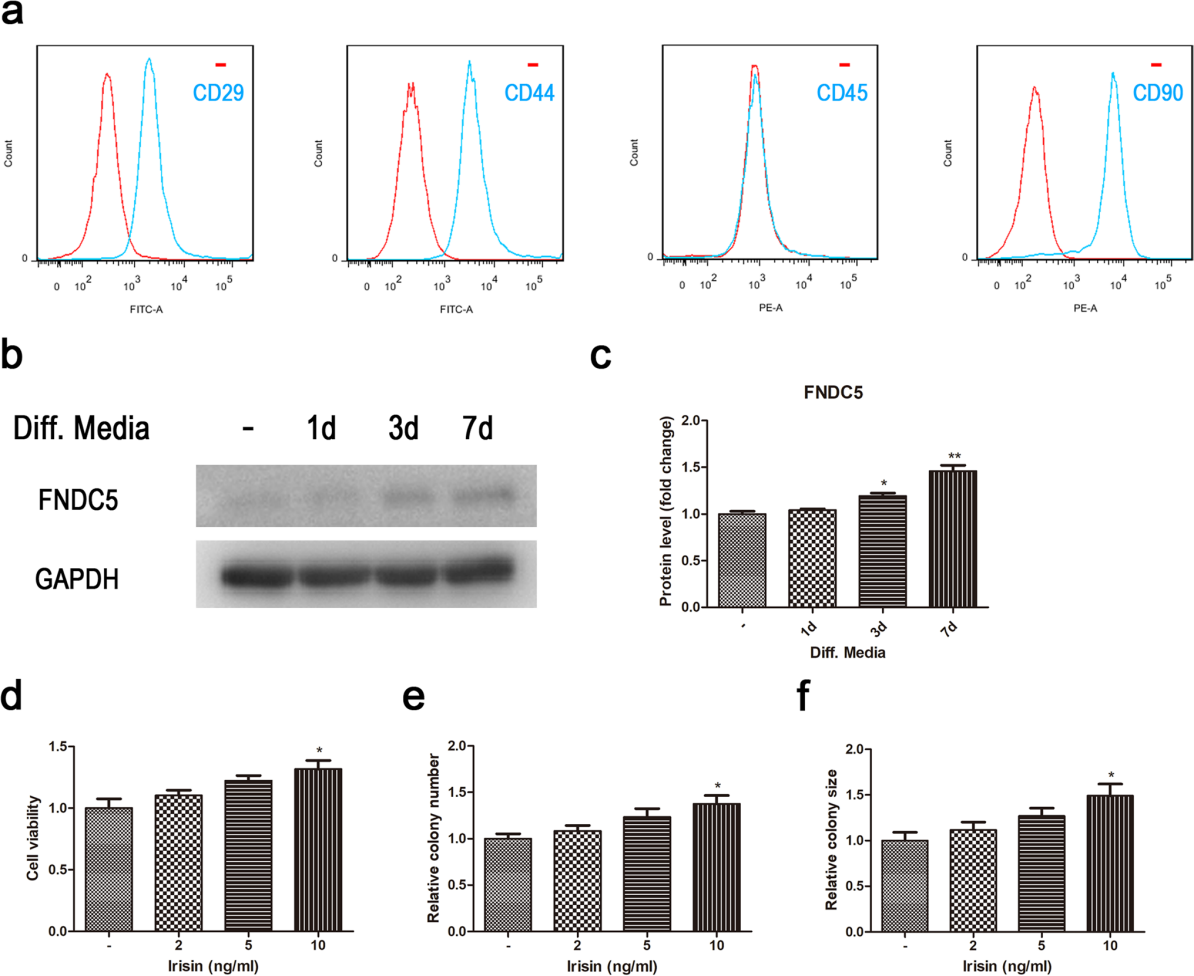
Effects of irisin on the proliferative and colony-forming abilities of rat TSPCs. (*a*) The cell surface markers analysis was performed to identify the stem status of cultured TSPCs using CD29, CD 44, CD45, and CD90 (red: control; blue: fluorescent antibody). (*b*–*c*) The FNDC5 protein expression levels of TSPCs in tenogenic differentiation induction medium. (*d*) Cell viability of TSPCs that treated with various concentrations of irisin (0, 2, 5, and 10ng/ml) for 3 d. (*e*) Relative colony number of TSPCs that treated with various concentrations of irisin (0, 2, 5, and 10ng/ml) for 3 d. (*f*) Relative colony size of TSPCs that treated with various concentrations of irisin (0, 2, 5, and 10ng/ml) for 3 d. All data are expressed as mean ± SD (*n* = 3). One-way ANOVA with a subsequent post hoc Tukey’s test was used for multiple comparisons. *p < 0.05, **p< 0.01 *versus* the blank group.

The cell surface markers analysis was performed to identify the stem status of the clonogenic cells. Results demonstrated that the clonogenic cells expressed the stem cell markers CD29, CD44, and CD99, but were negative for the leukocyte marker CD45 (Fig. 1*a*). To clarify if tenogenic differentiation could affect FNDC5/irisin expression and secretion in TSPCs, TSPCs were cultured in the tenogenic differentiation induction medium comprising high-glucose DMEM, 10% FBS, 37.5 μg/ml ascorbic acid, and 1% penicillin G for 7 d. The results of Western blot showed that the FNDC5 protein expression levels of TSPCs increased with the induction time (Fig. 1*b*–*c*). What’s more, we found that the secretion of irisin in TSPCs was obviously enhanced at day 1, and then it presented a decreasing tendency over the following 6-d period of the induced tenogenic differentiation (Figure S1). The cell viability assay and the colony formation assay were performed to evaluate the proliferative and colony-forming abilities of TSPCs treated with irisin of different concentrations (0, 2, 5, and 10ng/ml) for 3 d. The cell viability assay results showed that the relative cell viability of TSPCs increased with increasing concentrations of irisin (Fig. 1*d*). When compared to the blank group, irisin concentration of 10ng/ml could significantly increase the cell viability of TSPCs (*P*<0.05). The results indicated that irisin could increase the proliferative ability of TSPCs. Furthermore, the results of colony formation assay revealed that irisin did also play a positive role on the colony-forming ability of TSPCs. As shown in Fig. 1*e*–*f*, the relative colony number and size enlarged with irisin treatment in a dose-dependent manner, and significant difference was also observed at a concentration of 10ng/ml compared to the blank group. Taken together, these data confirmed the positive effects of irisin on the proliferative and colony-forming abilities of rat TSPCs.

### Involvement of irisin in tenogenic differentiation of rat TSPCs

**Figure 2.**
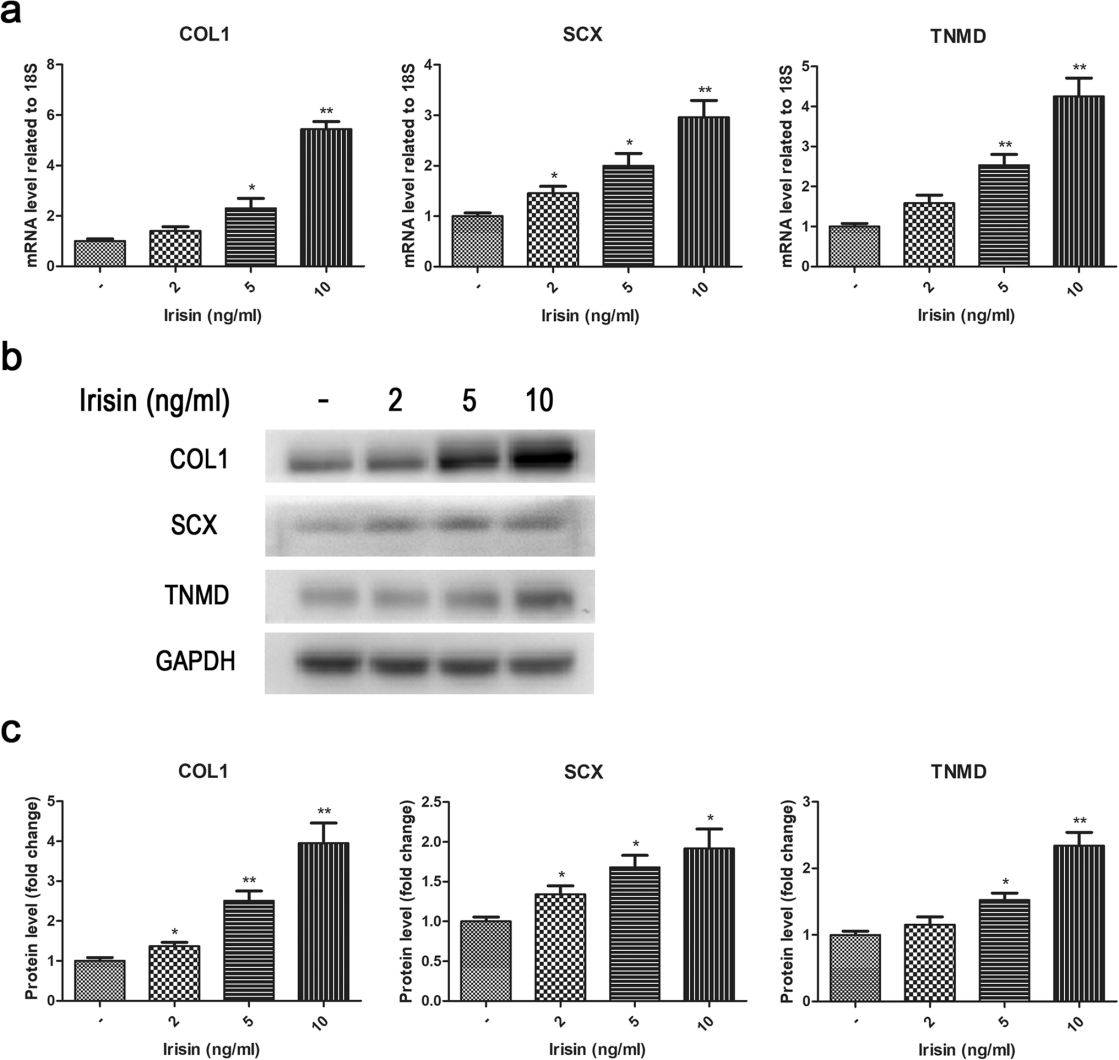
Effects of irisin on tenogenic differentiation of rat TSPCs. TSPCs were treated with various concentrations of irisin (0, 2, 5, and 10ng/ml) for 3 d. (***a***) The mRNA expression levels of extracellular matrix marker (COL1) and tenogenic markers (SCX, TNMD) were evaluated by RT-PCR. (***b***–*c*) The protein expression levels of extracellular matrix marker (COL1) and tenogenic markers (SCX, TNMD) were evaluated by Western blot. GAPDH as an internal control. All data are expressed as mean ± SD (*n* = 3). One-way ANOVA with a subsequent post hoc Tukey’s test was used for multiple comparisons. *p < 0.05, **p< 0.01 *versus* the blank group. COL1, type I collagen; SCX, scleraxis; TMND, tenomodulin.

TSPCs exhibit spontaneous tenogenic differentiation potentials in vitro (Shin *et al.* 2020; Liu *et al.* 2021). It has been recently demonstrated that the erroneous differentiation of TSPCs to non-tenocytes is involved in the pathogenesis of chronic tendinopathy (Lui and Chan 2011). Therefore, promoting differentiation of TSPCs into tenocytes under pathological conditions has been identified as an effective strategy for treatment of tendinopathy. To investigate the role of irisin on tenogenic differentiation of rat TSPCs, the mRNA and protein expression levels of tenogenic markers and extracellular matrix (ECM) markers were assessed by RT-PCR and Western blot. As illustrated in Fig. 2*a*, irisin could dose-dependently upregulate the mRNA expression of COL1, SCX, and TNMD. Moreover, the results of Western blot showed that the COL1, SCX, and TNMD protein expression levels also increased with irisin treatment in a dose-dependent manner (Fig. 2*b*–*c*). These results suggested that irisin could markedly promote tenogenic differentiation to some extent.

### Irisin regulated YAP/TAZ expression to promote tenogenic differentiation of rat TSPCs

**Figure 3.**
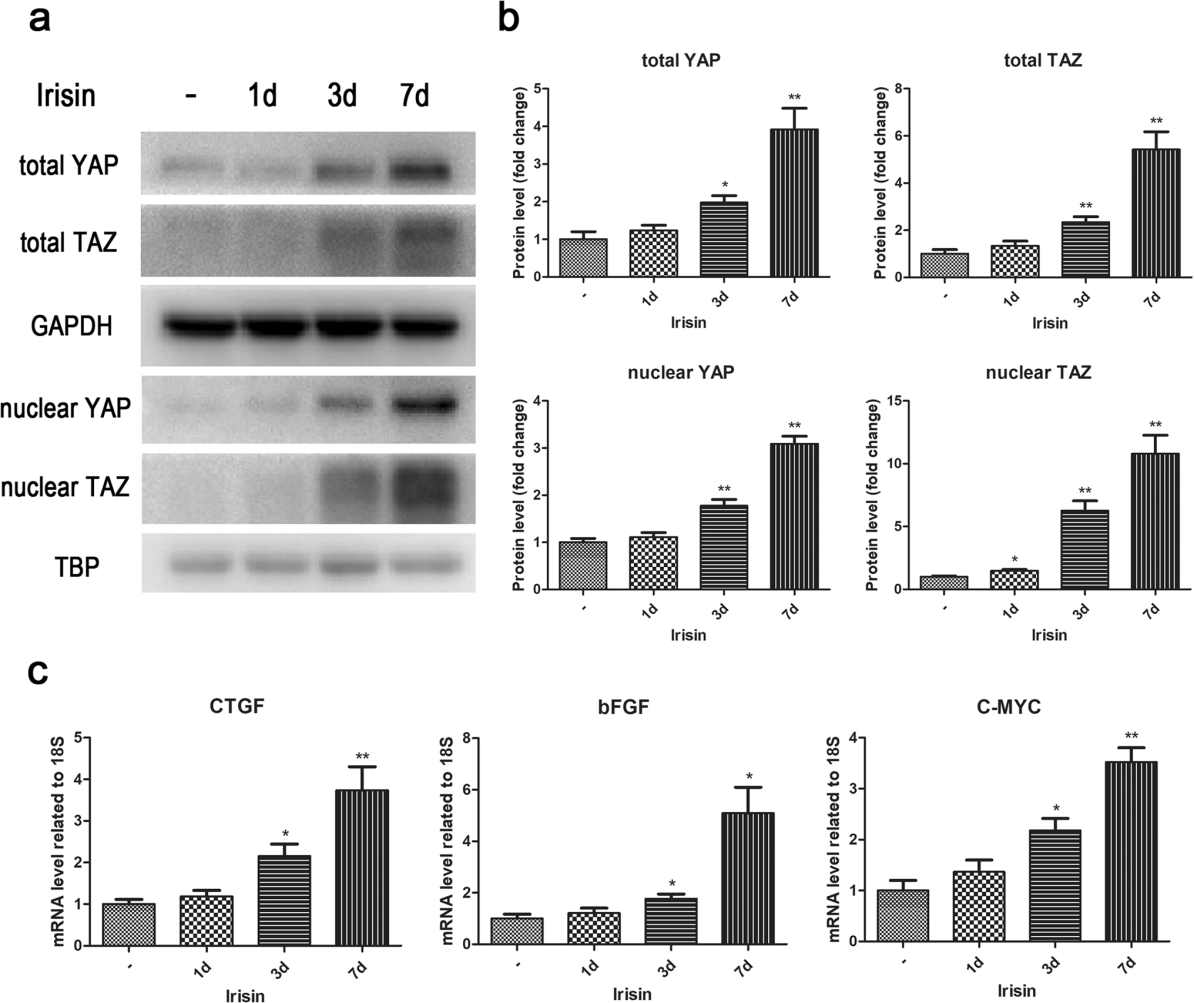
Effects of irisin on the expression of YAP/TAZ and the downstream target genes in rat TSPCs. TSPCs were treated with 10ng/ml irisin for various durations (0, 1, 3, and 7 d). (*a*–*b*) The protein expression levels of YAP, TAZ in the total cell lysate, as well as in the nucleus were evaluated by Western blot. GAPDH was used as an endogenous control in the cytoplasm, whereas TBP worked as an endogenous control in the nucleus. (*c*) The mRNA expression levels of CTGF, bFGF, and C-MYC were evaluated by RT-PCR. All data are expressed as mean ± SD (*n* = 3). One-way ANOVA with a subsequent post hoc Tukey’s test was used for multiple comparisons. *p < 0.05, **p< 0.01 versus the blank group. YAP, Yes-associated protein; TAZ, transcriptional co-activator with PDZ-binding motif; CTGF, connective tissue growth factor; bFGF, basic-fibroblast growth factor.

The role of the Hippo pathway and its downstream effectors, the transcriptional co-activators Yes-associated protein (YAP) and transcriptional co-activator with PDZ-binding motif (TAZ), on organ regeneration and regenerative medicine has attracted extensive attention of scholars in recent years (Moya and Halder 2019). To further clarify the detailed mechanism of irisin regulation of tenogenic differentiation, the influence of irisin on YAP/TAZ signaling in rat TSPCs was investigated. Protein levels of YAP, TAZ in the total cell lysate, as well as in the nucleus were assessed by Western blot. Results showed that, compared with the control group, the total and nuclear protein expression levels of YAP and TAZ were both significantly upregulated after treating with irisin for 3 d and 7 d (Fig. 3*a*–*b*). What’s more, the expression of downstream target genes was also detected by RT-PCR. As shown in Fig. 3*c*, the mediators of tenogenic induction (CTGF, bFGF) and proliferation promotion (C-MYC) mRNA levels were markedly upregulated by irisin treatment.

**Figure 4.**
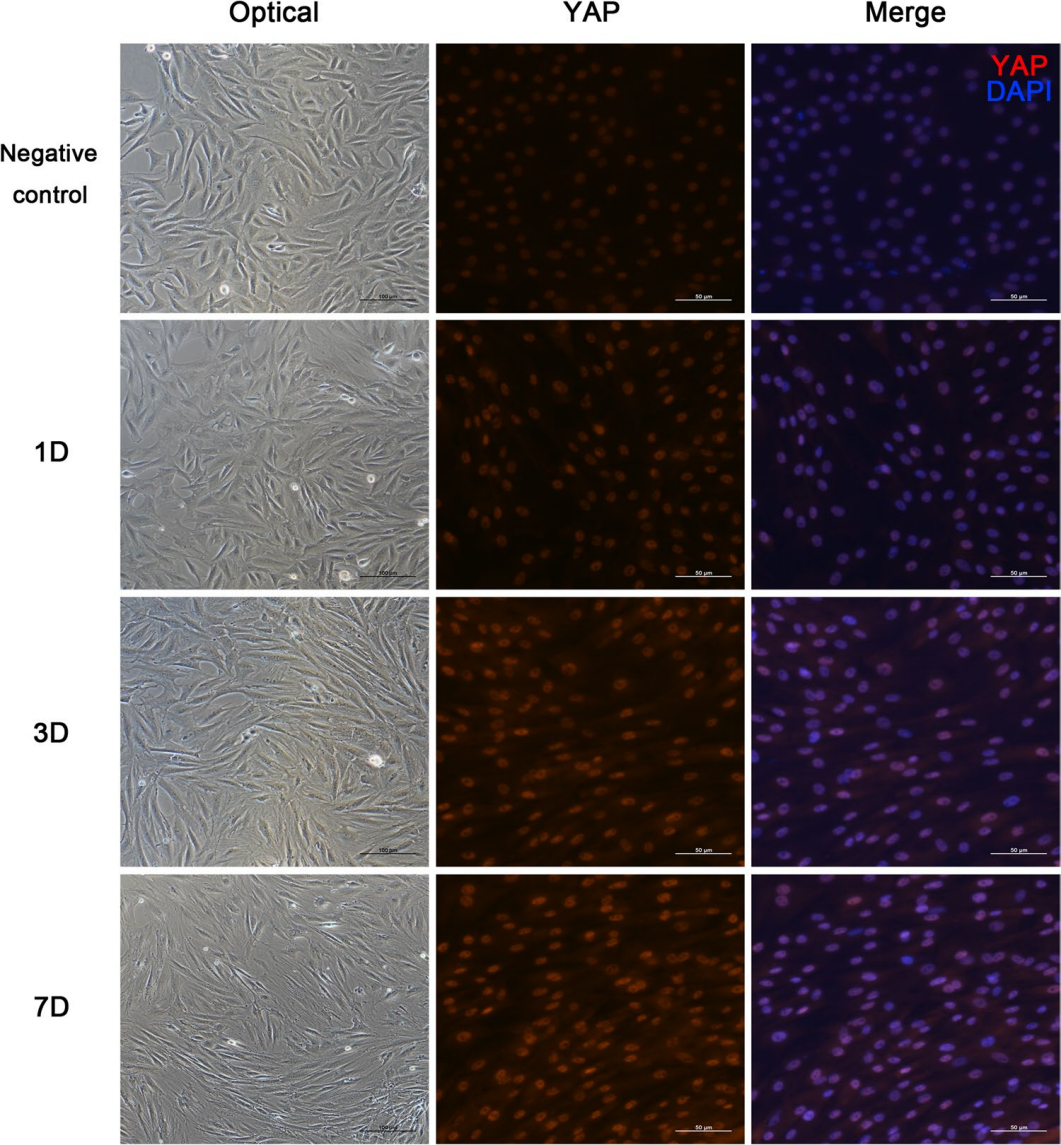
Effects of irisin on the morphological changes and subcellular positioning of YAP/TAZ in rat TSPCs. TSPCs were treated with 10ng/ml irisin for various durations (0, 1, 3, and 7 d). Optical morphological appearance of TSPCs, *bar* = 100μm. Immunofluorescence of YAP in TSPCs (*red*: YAP; *blue*: DAPI), *bar* = 50μm.

Previous studies have demonstrated that YAP/TAZ in the nuclear is crucial for the expression of target genes (Mo *et al.* 2014; Kwon *et al.* 2021; Ramos and Camargo 2012). Here, the morphological changes of treated TSPCs were observed by the optical microscopy, and the immunofluorescence staining of YAP in TSPCs incubated with 10ng/ml irisin for various times (0, 1d, 3d, 7d) was performed. TSPCs treated with irisin appeared slender and elongated, and the fluorescence intensity of cytoplasmic and nuclear YAP both increased with the duration of irisin treatment, while the enhanced YAP fluorescence signals were mainly observed in the nuclear of TSPCs (Fig. 4). To clarify why the protein expression levels of YAP were upregulated by irisin treatment, we further detected the effects of irisin on YAP mRNA expression levels by RT-PCR. The results demonstrated that the mRNA expression levels of YAP had no significant change after irisin treatment (Figure S2*a*). Since the overall protein levels of YAP/TAZ are known to be regulated by proteasomal degradation, we further investigated the role of irisin on baseline proteasome activity. Results suggested that irisin treatment reduced proteasome activity (Figure S2*b*). Together these results indicated that irisin treatment upregulated the protein expression levels of YAP/TAZ and this process was associated with a ubiquitin-proteasome proteolytic pathway.

**Figure 5.**
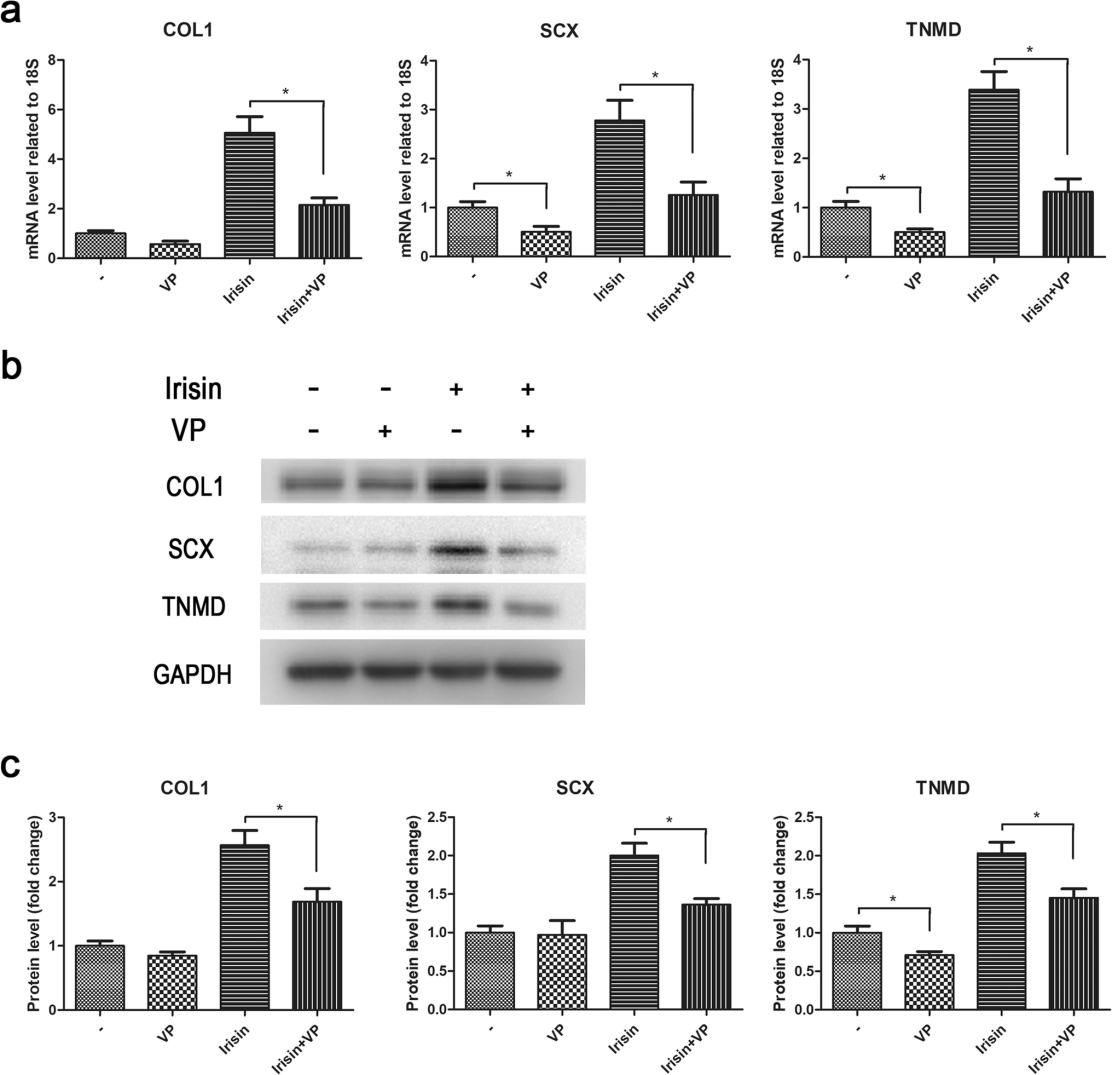
Effects of verteporfin on the irisin-promoted tenogenic differentiation of rat TSPCs. TSPCs were untreated or treated with 10ng/ml irisin or treated with 5μM verteporfin or treated with both for 3 d. (*a*) The mRNA expression levels of COL1, SCX, and TNMD were evaluated by RT-PCR. (*b*–*c*) The protein expression levels of COL1, SCX, and TNMD were evaluated by Western blot. GAPDH as an internal control. All data are expressed as mean ± SD (*n* = 3). One-way ANOVA with a subsequent post hoc Tukey’s test was used for multiple comparisons. *p < 0.05, **p< 0.01. VP, verteporfin.

In order to further ascertain the involvement of YAP/TAZ in the irisin-promoted tenogenic differentiation of rat TSPCs, we subsequently performed the pathway inhibition experiments using verteporfin, the inhibitor of YAP. As shown in Fig. 5*a*, RT-PCR results revealed that the increments of mRNA expression levels of COL1, SCX, and TNMD caused by irisin were markedly abolished by verteporfin treatment. Meanwhile, the protein expression levels of COL1, SCX, and TNMD showed similar changes following administration of verteporfin according to the Western blot results (Fig. 5*b*–*c*). Taken together, these data demonstrated that irisin could promote tenogenic differentiation of rat TSPCs via activating YAP/TAZ.

## Discussion

Now available treatments of tendinopathy still only control the symptoms due to the poor understanding of tendinopathy pathogenesis. Current evidence has demonstrated that the depletion of stem cell pool and altered TSPCs fate might account for the pathogenesis of tendinopathy (Lui and Chan 2011; Rui *et al.* 2013). Therefore, stem cell–based therapy has gained more attention in recent years. New therapeutic strategies about stem cell–based therapy for tendon repair and regeneration are emerging all the time.

Many kinds of stem cells, such as embryonic stem cells (ESCs), induced pluripotent stem cells (iPSCs), and mesenchymal stem cells (MSCs), have been proved to be the cell sources for tendon repair due to their potential for differentiating into tenocytes. Many animal studies have verified the active effects of these stem cells on tendon healing (Chen *et al.* 2009; Xu *et al.* 2013; Docheva *et al.* 2015). Tendon-derived stem/progenitor cells (TSPCs) are derived from tendon tissue. Unlike other MSCs, TSPCs express two tendon lineage-related markers scleraxis (SCX) and tendomodulin (TNMD) in vitro, and can differentiate into tendon-like tissues when implanted in vivo (Zhang *et al.* 2016).

However, there is a risk of ectopic ossification and tumor formation after stem cell transplantation (Harris *et al.* 2004; Zhang *et al.* 2008), which may be attributable to the erroneous differentiation of implanted stem cells. Thus, promoting TSPCs to differentiate towards the tenogenic lineage before transplantation might be a good strategy for successful tendon regeneration and repair. Recently, administration of various exogenous factors to promote exogenous tenogenesis has been proven to be effective. Growth differentiation factors (GDFs), connective tissue growth factor (CTGF), platelet-rich plasma (PRP), and biomaterial scaffold engineering are promising factors to induce tenogenic differentiation of TSPCs in vitro (Zhang *et al.* 2016).

It is well accepted that exercise is an important life style to prevent a variety of disorders (Tu *et al.* 2020). Current tendinopathy management tends to support exercise-based tendon rehabilitation as the most evidence-based intervention (Malliaras 2017; Rio *et al.* 2017; Cardoso *et al.* 2019). However, the potential mechanisms and key mediators of the positive effects of exercise remain unclear. FNDC5/Irisin, as one of novel exercise-induced myokines, might be involved in the exercise-induced protective effects on tendinopathy.

In the present study, we analyzed the effects of irisin on the proliferation and tenogenic differentiation of TSPCs, focusing on the phenotypic changes of TSPCs and the underlying mechanism. Our study firstly identified the TSPCs by the immunophenotype (CD29(+), CD44(+), CD90(+), and CD45(−)), and it was observed that the protein expression levels of FNDC5 in TSPCs contiguously increased in a time-dependent manner over a 7-d period of the tenogenic differentiation. Interestingly, the secretion of irisin in TSPCs presented a decreasing tendency after day 1. Irisin, as the secreted form of FNDC5, is proteolytically cleaved and secreted in response to physical stimulation. It is likely that while the protein expression levels of FNDC5 in TSPCs increased during the tenogenic differentiation, the cleavage of irisin from FNDC5 and secretion from TSPCs was not affected. To evaluate whether irisin could directly regulate the TSPC tenogenic differentiation process, the tenogenic markers and extracellular matrix (ECM) markers were assessed. We found that the mRNA and protein expression levels of COL1, SCX, and TNMD upregulated with increasing irisin concentration. What’s more, in line with previous studies (Würgler-Hauri *et al.* 2007; Chen *et al.* 2008), increased CTGF mRNA expression was observed during the irisin-induced TSPC tenogenic differentiation process in our study. Besides, we also found the upregulated mRNA expression of another tenogenic induction gene (bFGF) and proliferation promotion gene (C-MYC) in TSPCs during irisin treatment, which might also contribute to the irisin-induced proliferation and tenogenic differentiation of TSPCs.

The Hippo pathway has been proven to play crucial roles in the regulation of stem cell pluripotency/differentiation (Ramos and Camargo 2012; Mo *et al.* 2014). The Hippo pathway can be regulated by various factors, such as cellular stresses, mechanical stimuli, and biochemical signals (Kwon *et al.* 2021). YAP and TAZ are essential transcriptional co-activators and downstream effectors of the Hippo pathway, regulating the expression of target genes related to cell proliferation, tissue homeostasis, and tissue regeneration (Lin *et al.* 2017). In our study, we found that irisin treatment could upregulate total and nuclear protein levels of YAP/TAZ, and further promoted the expression of downstream target genes (CTGF, bFGF, and C-MYC). Verteporfin, the inhibitor of YAP, was found to reverse the effect of irisin on TSPCs. These results indicated that YAP/TAZ signaling participated in the process of irisin-induced tenogenic differentiation of TSPCs.

However, there are still some limitations in our study. Firstly, we confirmed the positive effect of irisin on TSPC proliferation and tenogenic differentiation in vitro via upregulating YAP/TAZ protein expression. But the effect of irisin on tendon healing in vivo should be further investigated. Secondly, there is a large difference between the circulating irisin concentrations in rat and human, so the role of irisin on human cells should be further evaluated. Last but not the least, in order to explore the precise exercise treatment program for individuals, the release mode of irisin by muscle cells when responding to exercise needs to be further investigated.

## Conclusions

In summary, our findings indicated that irisin promoted the proliferation and tenogenic differentiation of rat TSPCs in vitro by activating YAP/TAZ. Therefore, our study provides a novel strategy for tendon repair. Irisin may be a powerful exercise-induced agent for tendinopathy treatment, and YAP/TAZ could be a promising target.

## Supplementary information

Fig. S1 Effects of tenogenic differentiation induction medium on irisin secretion in rat TSPCs. TSPCs were cultured in the tenogenic differentiation induction medium for 7 d, and the medium was changed every day. The irisin concentrations of cell culture supernates at day 1, 3, and 7 were evaluated by ELISA. *p < 0.05 versus the blank group. (PNG 19 kb)

High Resolution (TIF 53 kb)

Fig. S2 Effects of irisin on the mRNA expression levels of YAP and proteasomal degradation in rat TSPCs. TSPCs were treated with 10ng/ml irisin for various durations (0, 1, 3, and 7 d). (a) The mRNA expression levels of YAP were evaluated by RT-PCR. (b) The chymotrypsin-like activity of the 26S proteasome was evaluated via cell-based luminescent assay. *p < 0.05 versus the blank group. (PNG 387 kb)

High Resolution (TIF 655 kb)
