# Supplementary figures and images for: Irisin promotes the proliferation and tenogenic differentiation of rat tendon-derived stem/progenitor cells via activating YAP/TAZ
Source: In Vitro Cell Dev Biol Anim. 2022 Sep 20;58(8):658–68. doi: 10.1007/s11626-022-00699-2 (PMC9550707; doi:10.1007/s11626-022-00699-2)

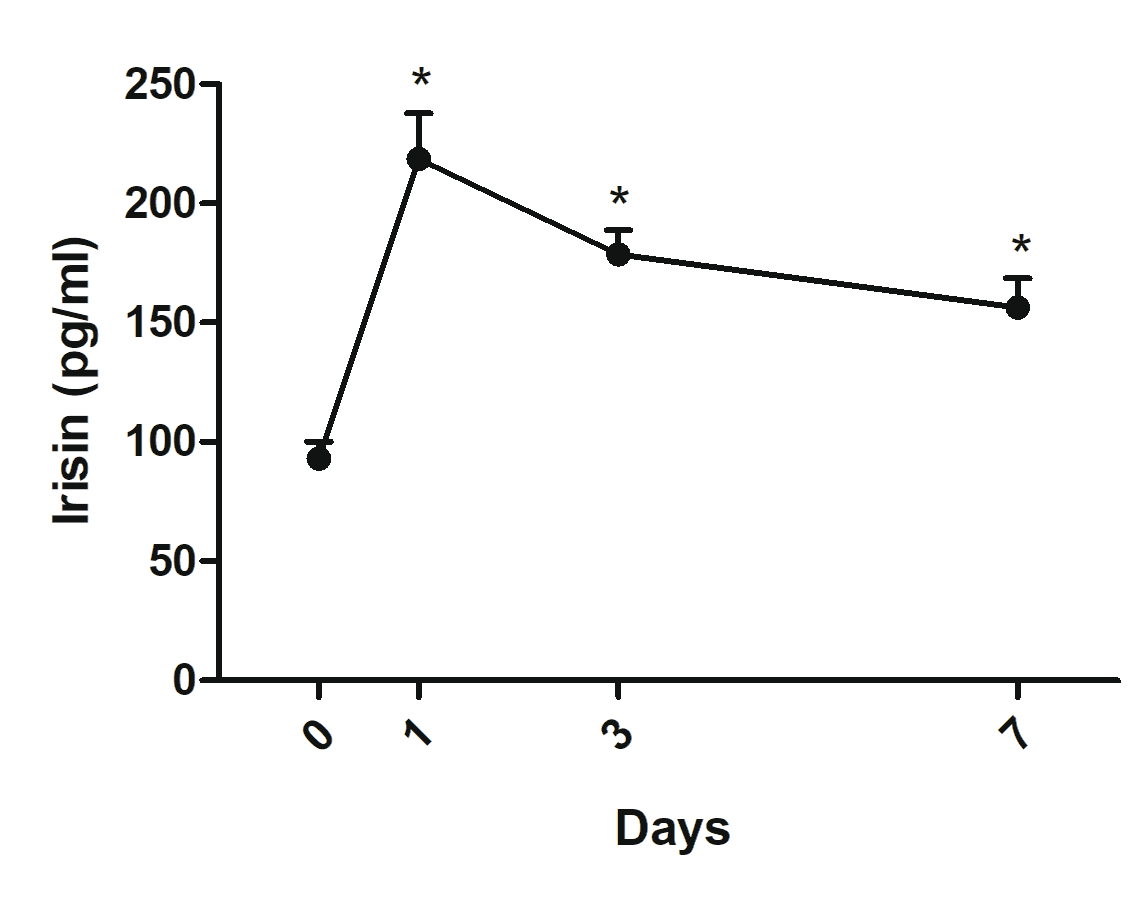

Supplement: Supplementary file 1 — Effects of tenogenic differentiation induction medium on irisin secretion in rat TSPCs. TSPCs were cultured in the tenogenic differentiation induction medium for 7 d, and the medium was changed every day. The irisin concentrations of cell culture supernates at day 1, 3, and 7 were evaluated by ELISA. *p < 0.05 versus the blank group. (PNG 19 kb) [file 11626_2022_699_Fig6_ESM.png]

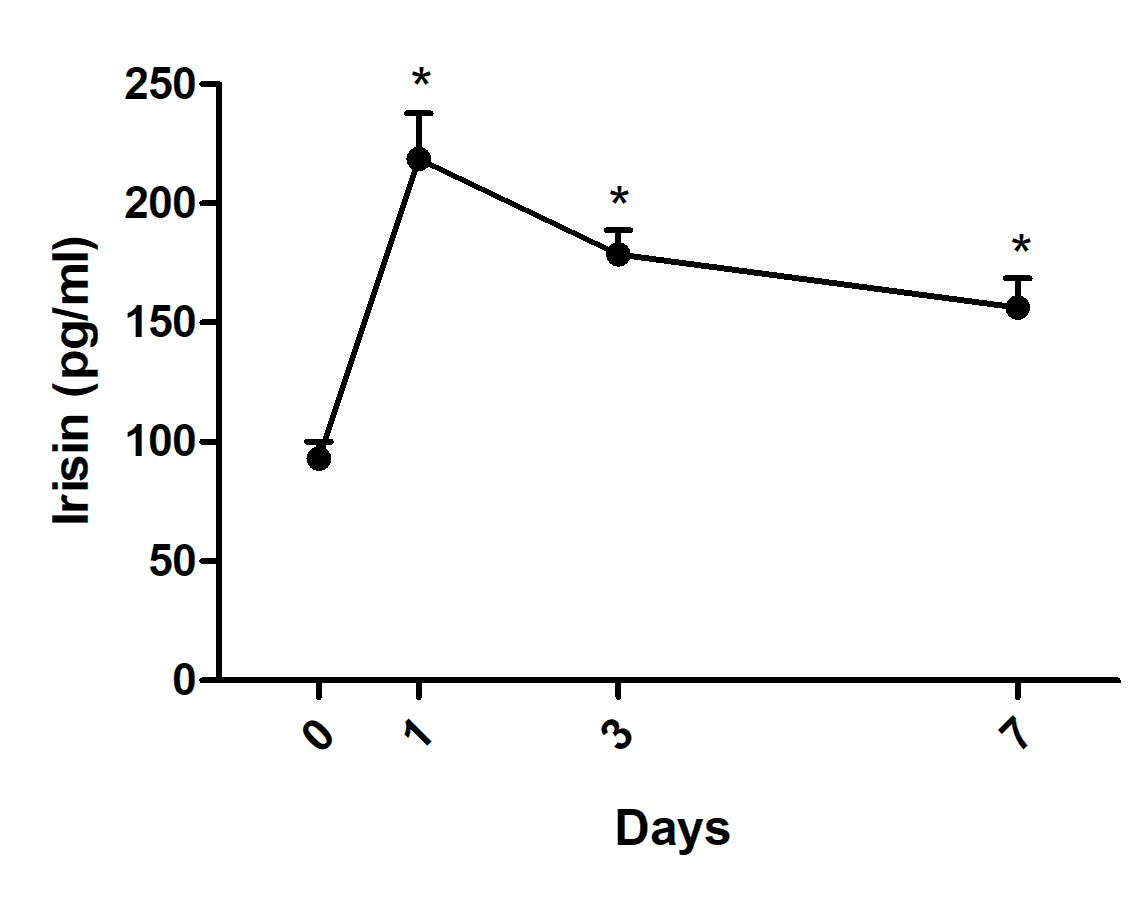

Supplement: Supplementary file 2 — High Resolution (TIF 53 kb) [file 11626_2022_699_MOESM1_ESM.tif]

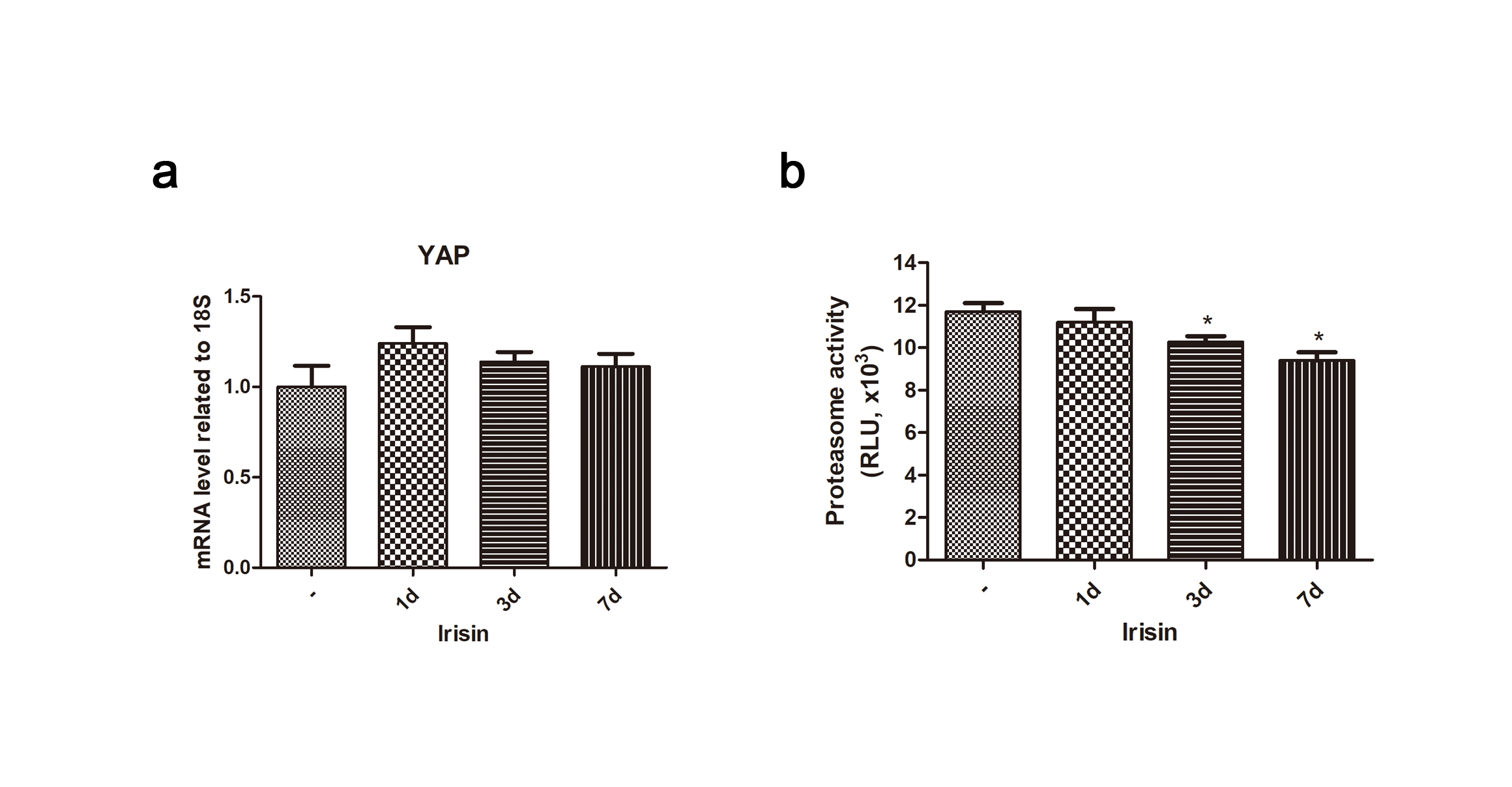

Supplement: Supplementary file 3 — Effects of irisin on the mRNA expression levels of YAP and proteasomal degradation in rat TSPCs. TSPCs were treated with 10ng/ml irisin for various durations (0, 1, 3, and 7 d). (a) The mRNA expression levels of YAP were evaluated by RT-PCR. (b) The chymotrypsin-like activity of the 26S proteasome was evaluated via cell-based luminescent assay. *p < 0.05 versus the blank group. (PNG 387 kb) [file 11626_2022_699_Fig7_ESM.png]

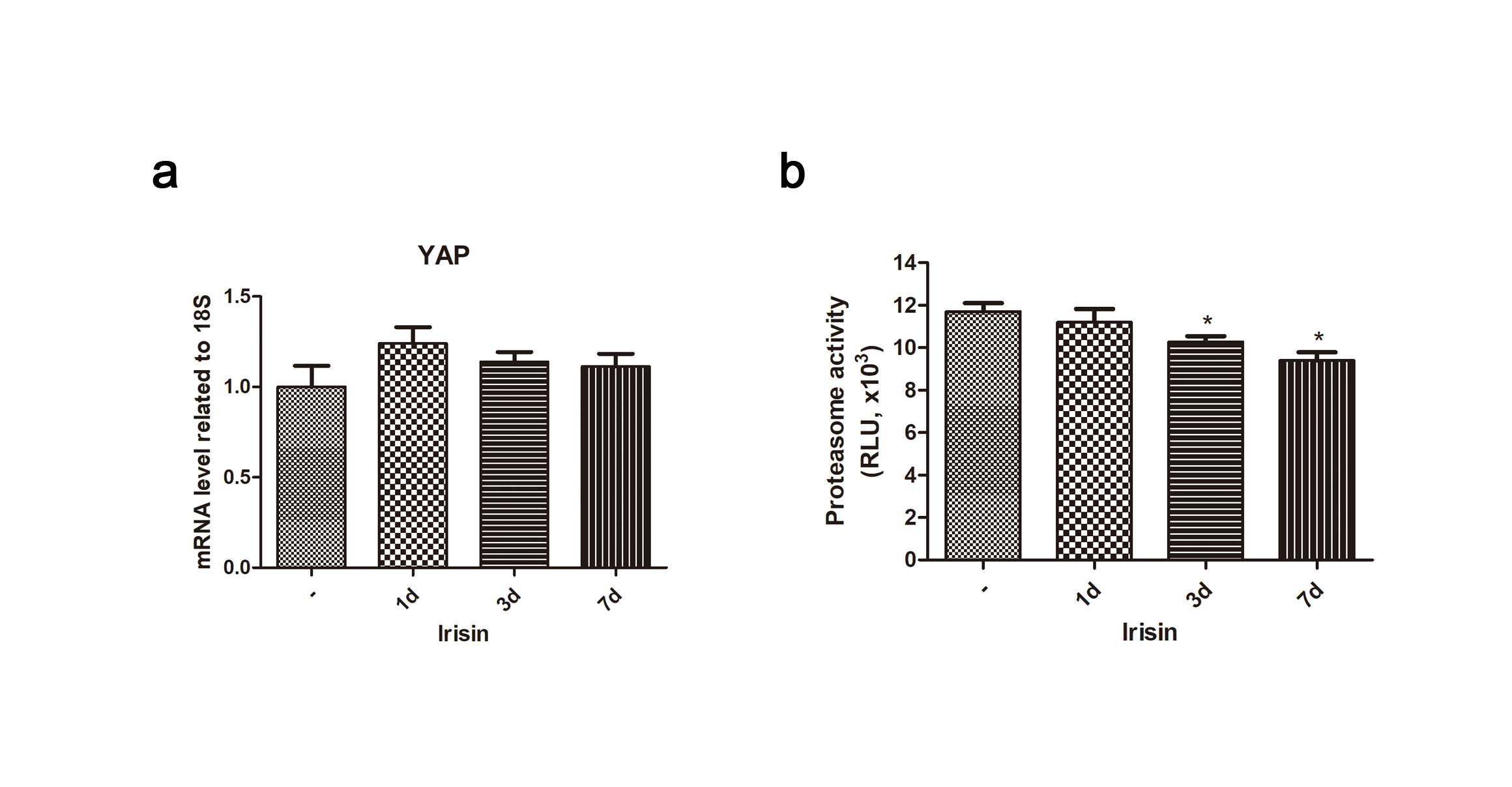

Supplement: Supplementary file 4 — High Resolution (TIF 655 kb) [file 11626_2022_699_MOESM2_ESM.tif]
